# Microbial Community Characterizing Vermiculations from Karst Caves and Its Role in Their Formation

**DOI:** 10.1007/s00248-020-01623-5

**Published:** 2020-11-06

**Authors:** Rosangela Addesso, Jose L. Gonzalez-Pimentel, Ilenia M. D’Angeli, Jo De Waele, Cesareo Saiz-Jimenez, Valme Jurado, Ana Z. Miller, Beatriz Cubero, Giovanni Vigliotta, Daniela Baldantoni

**Affiliations:** 1 Department of Chemistry and Biology “Adolfo Zambelli”, University of Salerno, Via Giovanni Paolo II, 132, 84084 Fisciano, SA Italy; 2 HERCULES Laboratory, University of Évora, Largo Marques de Marialva 8, 7000-809 Évora, Portugal; 3 Department of Biological, Geological and Environmental Sciences, University of Bologna, Via Zamboni, 67, 40126 Bologna, Italy; 4 Instituto de Recursos Naturales y Agrobiología de Sevilla, IRNAS-CSIC, Av. Reina Mercedes, 10, 41012 Sevilla, Spain

**Keywords:** Vermicular deposits, Underground ecosystem, Geomicrobiology, Cave ecology, Next-generation sequencing, Pertosa-Auletta Cave

## Abstract

The microbiota associated with vermiculations from karst caves is largely unknown. Vermiculations are enigmatic deposits forming worm-like patterns on cave walls all over the world. They represent a precious focus for geomicrobiological studies aimed at exploring both the microbial life of these ecosystems and the vermiculation genesis. This study comprises the first approach on the microbial communities thriving in Pertosa-Auletta Cave (southern Italy) vermiculations by next-generation sequencing. The most abundant phylum in vermiculations was *Proteobacteria*, followed by *Acidobacteria* > *Actinobacteria* > *Nitrospirae* > *Firmicutes* > *Planctomycetes* > *Chloroflexi* > *Gemmatimonadetes* > *Bacteroidetes* > *Latescibacteria*. Numerous less-represented taxonomic groups (< 1%), as well as unclassified ones, were also detected. From an ecological point of view, all the groups co-participate in the biogeochemical cycles in these underground environments, mediating oxidation-reduction reactions, promoting host rock dissolution and secondary mineral precipitation, and enriching the matrix in organic matter. Confocal laser scanning microscopy and field emission scanning electron microscopy brought evidence of a strong interaction between the biotic community and the abiotic matrix, supporting the role of microbial communities in the formation process of vermiculations.

**Supplementary Information:**

The online version contains supplementary material available at 10.1007/s00248-020-01623-5.

## Introduction

The hypogean environments are the least known and studied on Earth [1]. Despite the prohibitive abiotic factors (e.g., oligotrophy, total darkness, and high mineral concentrations) for life development, they represent interesting ecological niches, hosting extremophile microorganisms, highly specialized and perfectly adapted to this peculiar ecosystem, showing an unexpected biodiversity within the *Bacteria* domain and countless novel species [2]. To overcome the limiting factors, microorganisms create mutualistic networks, cooperating in communities and favoring each other’s survival. The autotrophic microorganisms generally draw energy by chemosynthesis, using chemical elements (such as Ca, Mg, Fe, Mn, and S) and organic and inorganic compounds abundant in the host rocks, cave sediments, groundwater, and atmosphere. Concurrently, several microbial groups rely on mixed metabolic pathways (mixotrophy) [3]. In any case, such microbial communities may contribute to the formation of caves, influencing several biogeochemical processes [1, 4–7]. In particular, they act inducing the precipitation [8, 9] or dissolution of minerals of speleothems and other structures occurring in underground environments, like moonmilk and vermiculation deposits [10, 11]. The genesis of all these examples is, indeed, difficult to be explained only by pure abiotic physicochemical processes [2].

Among cave structures, vermiculations are enigmatic deposits recurring on rock surfaces in caves all over the world [12–14], characterized by variable morphologies, colors, and dimensions [15, 16], and generally composed of calcite, associated with quartz, and traces of clay minerals [17]. Recent studies highlighted microbial evidences supporting their biological origin [10, 17–19]. Vermiculations can be indeed considered “life hotspots” and a precious support for the studies on cave geomicrobiology. To our knowledge, there are still few studies on their microbial characterization and most of these concern vermiculations from sulfuric acid speleogenetic systems [10, 20, 21].

Aimed at shedding light on the microbial community of vermiculations from the Pertosa-Auletta Cave (Campania, southern Italy) and on its role in their formation, this work represents one of the first microbiological studies of vermicular deposits from a normal epigenic karst system. To this end, molecular biology approaches have been employed. In addition, giving an important contribution to the knowledge of the hidden biological aspects of vermiculations, it represents a key step toward the protection and conservation of these peculiar biosignatures and of the whole cave ecosystem.

## Methods

### Vermiculation Samplings

**Fig. 1:**
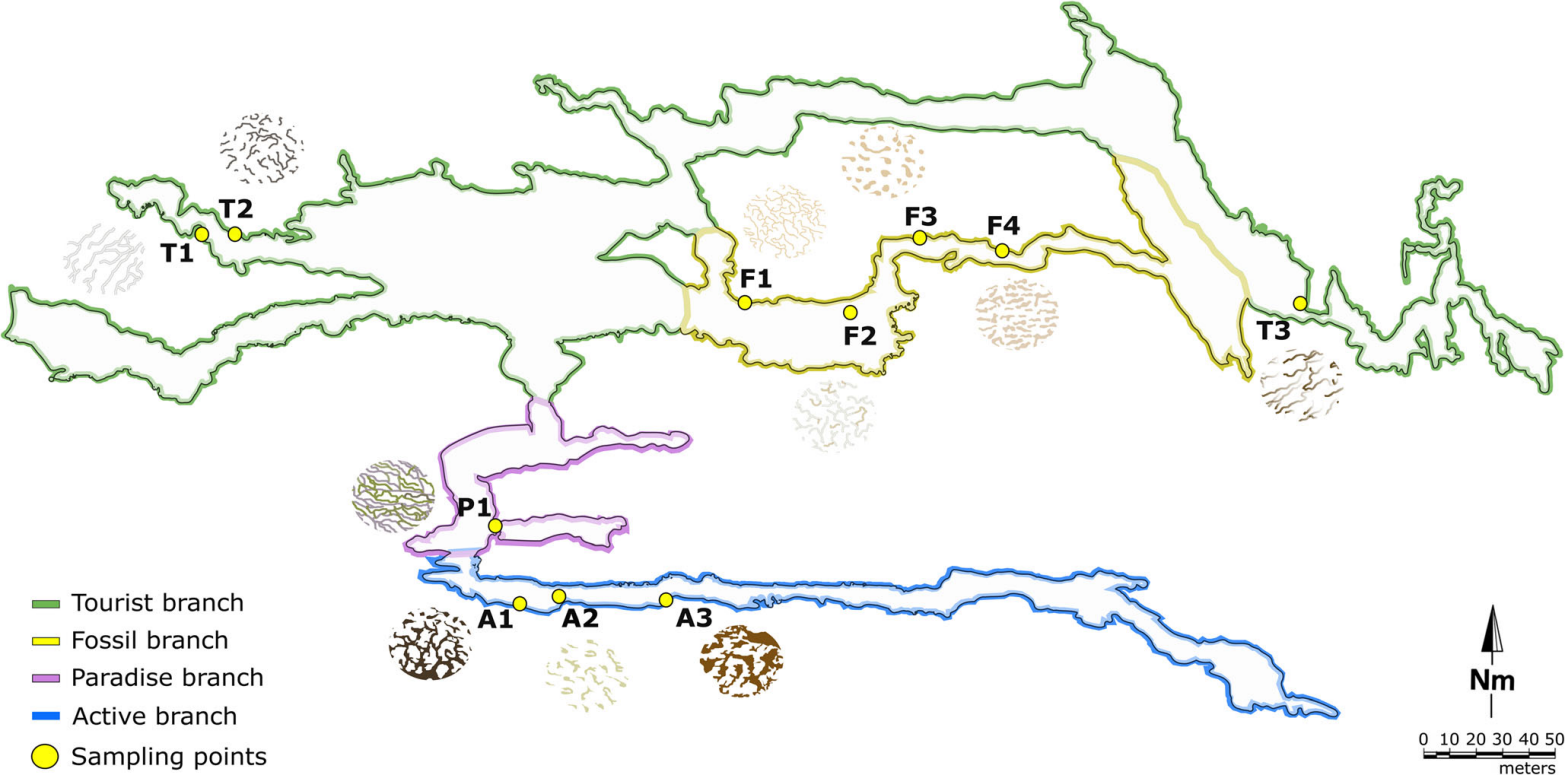
Pertosa-Auletta Cave (Campania, southern Italy) karst system; yellow points indicate the collected vermiculations, with the corresponding texture model, in the Active (A, blue), Fossil (F, yellow), Paradise (P, violet), and Tourist (T, green) trails

Eleven different points were sampled in the four main branches of the Pertosa-Auletta Cave (Fig. 1), a limestone show cave in southern Italy. Approximately, 2 g of vermiculation deposits was collected. The four branches of the studied karst cave are characterized by various degrees of frequentation, namely (i) Active (A), (ii) Fossil (F), (iii) Paradise (P), and (iv) Tourist (T), where Active indicates the branch still influenced by an active water flow, Fossil identifies inactive conditions of water flow, Paradise is a short piece of the active branch, lit and frequented by humans, and Tourist is the illuminated trail opened to the public for regular visits.

An accurate description of the study area, as well as on the geochemistry of the vermiculations, is reported in Addesso et al. [17]. In particular, the 11 samples of vermicular deposits, described in detail for their morphology, color, chemical and mineralogical composition [17], showed several shapes as described by Parenzan [15, 22] classification and can be divided into hieroglyphic (A1, A2, A3, F2, T2), dendritic (F1, P1, T1), bubble-like spots (F3), large-leopard spots (F4), and tiger skin (T3). Colors ranged from whitish (A2, F1, T2) to grey (P1, T2) or brown (A1, A3, F1, F3, F4, T3), greenish in P1, probably due to the presence of photoautotrophs [17].

The sampling was performed using disposable and sterile scalpel blades and Eppendorf tubes, carefully avoiding damage to the walls. Stored at 4 °C, the samples were immediately sent to the Instituto de Recursos Naturales y Agrobiologia of Sevilla (IRNAS-CSIC, Spain) and maintained at − 80 °C, until processing.

### Molecular Analyses

Total DNA was extracted using FastDNA^TM^ Spin Kit for Soil, according to the producer’s protocol (MP Biomedical). The DNA quality was determined by a Nanodrop ND-1000 Spectrophotometer, whereas the amount by a Qubit 2.0 Fluorometer (Invitrogen).

Prokaryotic 16S and eukaryotic 18S rRNA genes were amplified by polymerase chain reaction (PCR), using specific primers: 616F [23] and 1510R [24] for *Bacteria*, 109F and 915R [25] for *Archaea*, EukA and EukB [26] for *Eukarya*, ITS1 and ITS4 [27] for *Fungi*. PCR reactions were carried out using 0.2-mL PCR tubes with a minimal amount of extracted DNA (from 0.5 to 2.0 μL), pure and diluted to 2 and 5 ng/μL, and 50 μL of Mastermix solution [1 mL = 775 μL H_2_O(σ), 200 μL of PCR Buffer (BIOLINE) and 5 μL Taq Polymerase (BIOLINE), 10 μL specific primers (Reverse and Forward), 4 μL BSA 10%], employing a FlexCycler (Analytik Jena) and a T100 Thermal Cycler (Bio-Rad). The PCR thermal programs are given in Table S1. The amplified PCR products underwent 1% agarose gel electrophoresis (0.5 M TAE Buffer) for a qualitative analysis. Fingerprints of *Archaea* and *Bacteria* communities were obtained by denaturing gradient gel electrophoresis (DGGE) of samples, according to Muyzer et al*.* [28], using a DCODE™ System (Bio-Rad).

The extracted DNA (with a minimum concentration of ~ 5 ng/μL), after purification by Genomed and Genomic DNA Clean & Concentrator™-10 (Zymo Research), was analyzed by via next-generation sequencing (NGS) targeting the V3–V4 hypervariable region of Prokaryotes 16S rRNA, using Illumina MiSeq 2 × 250 paired end, according to Macrogen (Seoul, Korea) library preparation protocol. Chimeras were identified and removed by means of USEARCH [29]. Resulting reads were processed in Qiime [30], whereas UCLUST [29] was used for the similar sequences assignment to operational taxonomic units (OTUs) by clustering with a 97% similarity threshold. Paired-end reads were merged using FLASH [31]. RDP Release 11 was used as against reference database for taxonomic identification of query sequences. Alpha diversity analysis, including estimation of Chao1, Shannon, Simpson, and Good’s Coverage indices, and rarefaction curves, based on the observed species metric, were performed through Qiime.

The graphs relative to molecular analysis data were elaborated in the R 3.6.0 programming environment [32]. The barplots, showing the relative abundances at phylum, class, and order levels for each sample, with associated dendrograms explaining the similarities among the samples, were created using “ggplots2”, “dendextend”, and “RColorBrewer” packages. Pearson’s correlation coefficients (*r* values) were obtained using cor function to evaluate associations (for *α* = 0.05) between geochemical characteristics and microbial phyla as well as among biological properties of the analyzed vermiculations. Non-metric multidimensional scaling (NMDS) analysis, with superimposition of confidence ellipses for branches (*α* = 0.05), and principal component analysis (PCA) were performed using meta.mds function, based on Euclidean distance metric, and prcomp function, respectively, both from “vegan” package.

### Microscopy

The nucleic acids of the whole cells were visualized using the specific SYBR Green fluorescent dye (1:100 dilution), on samples not handled further, under an Olympus FluoView FV1000 confocal laser scanning microscope, and the 488-nm excitation laser line with emission signal being collected at 510–530 nm. Images were analyzed with the FluoView 2.1 software (Olympus). FESEM images were acquired using FEI Teneo (Thermo Fisher, MA, USA). To this end, samples were prepared as reported in Addesso et al. [17]. In particular, they were fixed with 2.5% glutaraldehyde in 0.1 M cacodylate buffer (pH 7.4) at 4 °C for 2 h and washed thrice in cacodylate buffer. Subsequently, they were treated with 1% osmium tetroxide for 1 h at 4 °C and dehydrated by subsequent dilution series in ethanol and acetone finishing with 100% acetone before drying. The samples were dried in a EM CPD 300 (Leica Microsystem, Wetzlar, Germany) critical point drying device at 34.5 °C. Finally, samples were mounted on SEM stubs and sputter-coated with gold (5–10 nm).

## Results

All the 11 studied vermiculations, developing on limestone substratum (except A1 and A3, in the Active branch, which were growing at the interface between limestone host rock and bat guano crusts), showed a considerable biological diversity.

### Taxonomic Composition of Microbial Community

The preliminary qualitative analysis on the DNA extracted from vermiculations gave positive results for Prokaryotes and negative results for Eukaryotes. Online Resource 1 displays the archaeal (a) and bacterial (b) 16S rRNA gene-DGGE profiles of the sampled vermiculations. NGS analysis of 16S rRNA gene identified archaeal and bacterial taxa. *Archaea* were scarcely represented (Table 1). At the phylum level, *Thaumarchaeota* was characterized in all the vermiculations, with a relative abundance varying between 0.01 and 0.07%: *Woesearchaeota* was present in all the samples (0.01–0.04%), except for A2, P1, and T3, whereas *Euryarchaeota* was detected in F3 (0.01%) and P1 (0.03%). Moreover, unclassified *Archaea* were found in A3, F1, F2, F3, P1, T1, and T2 in percentages ranging from 0.01 and 0.09% (Table 1).

**Table 1:** Relative abundance (%) of *Archaea* at phylum level for each vermiculation sample

Phylum	A1	A2	A3	F1	F2	F3	F4	P1	T1	T2	T3
Unclassified	–	–	0.01	0.04	0.08	0.05	–	0.01	0.09	0.02	–
*Euryarchaeota*	–	–	–	–	–	0.01	–	0.03	–	–	–
*Thaumarchaeota*	0.01	0.06	0.06	0.05	0.07	0.05	0.05	0.03	0.07	0.05	0.06
*Woesearchaeota*	0.01	–	0.01	0.03	0.03	0.02	0.01	–	0.04	0.02	–

**Fig. 2:**
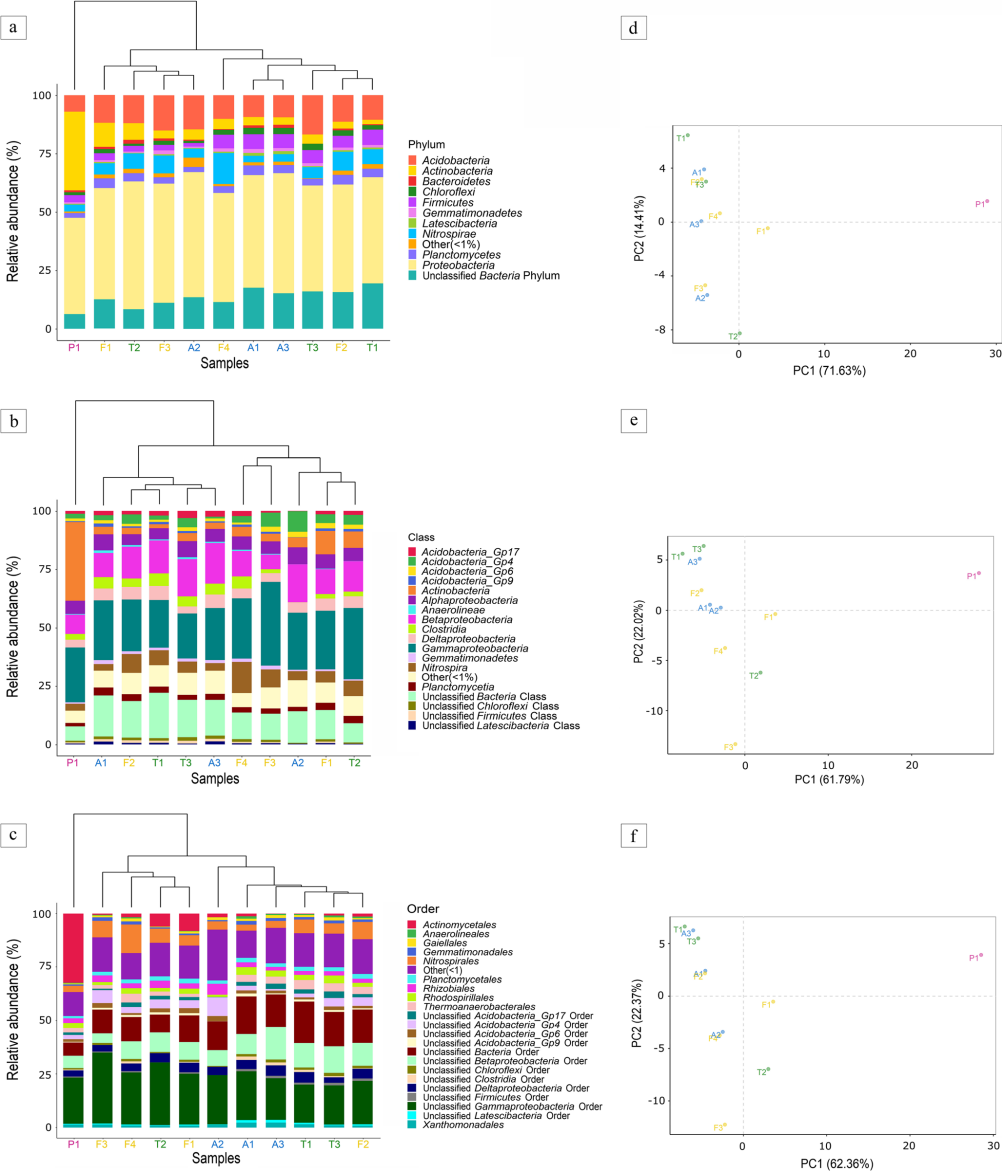
Bacterial composition of vermiculations from Pertosa-Auletta Cave; the barplots show the relative abundances (%) at phylum (**a**), class (**b**), and order (**c**) levels of samples from the Active (A, blue), Fossil (F, yellow), Paradise (P, violet), and Tourist (T, green) branches, with corresponding dendrograms (**a**, **b**, **c**) and PCA analysis (**d**, **e**, **f**)

*Bacteria* composed almost the entire extracted DNA (Fig. 2). The major phylum in the total bacterial community was *Proteobacteria* (41.3–54.8%), followed by *Acidobacteria* (7.1–16.8%) > *Actinobacteria* (1.9–33.8%) > *Nitrospirae* (2.8–13.3%) > *Firmicutes* (1.5–6.6%) > *Planctomycetes* (2.0–4.2%) > *Chloroflexi* (0.9–2.7%) > *Gemmatimonadetes* (0.6–1.7%) > *Bacteroidetes* (0.04–1.7%) > *Latescibacteria* (0.2–1.3%). NGS analysis highlighted the presence of a very copious group of unclassified phyla with percentage ranging from 6.2 and 19.3%. Other 16 phyla were less represented (< 1%). The microbial abundances were very similar in all the vermiculations, except P1, dominated by *Actinobacteria* (33.8%) in addition to *Proteobacteria* (41.3%) (Fig. 2a). The most abundant classes within the *Proteobacteria* phylum were as follows: *Gamma-* (19.3–35.8%) > *Beta-* (6.3–17.4%) > *Alpha-* (4.6–7.2%) > *Delta-* (3.3–5.9%) (Fig. 2b). At the order level (Fig. 2c), *Gammaproteobacteria* was mainly represented by an ample unclassified group (17.7–33.1%) and by *Xanthomonadales* (< 2.3%), whereas *Alphaproteobacteria* included the *Rhizobiales* (1.7–5.1%) and *Rhodospirillales* (1.1–3.6%) orders. *Nitrospirales* > *Actinomycetales* > *Thermoanaerobacterales* > *Planctomycetales* > *Gemmatimonadales* > *Gaiellales* > *Anaerolineales* were also identified with an abundance below 5.9%. Numerous unclassified groups were present at the order level, increasing considerably in the subsequent taxonomic levels.

The dendrograms (Fig. 2), showing similarities and divergences between specimens based on taxon relative abundances, highlighted three groups, keeping enough in the graph representations of all three taxonomic levels. The clustering analysis showed a clear separation of P1, the only sample located in Paradise branch, from the other two groups, closer to each other (Fig. 2a–c). At the phylum level (Fig. 2a), A2, F1, F3, and T2 clustered together from the rest (A1, A3, F2, F4, T1, T3). At the class level (Fig. 2b), F2 grouped with A2, F1, F3, and T2, splitting up from A1, A3, F4, T1, and T3. Lastly, at the order level (Fig. 2c), F1, F3, F4, and T2 assembled a new cluster divided from the remaining samples. Figure 2 also shows the corresponding PCAs based on the total bacterial communities at the phylum (Fig. 2d), class (Fig. 2e), and order (Fig. 2f) levels. Analogous clusters of the dendrograms were also observed in PCA plots. The first (PC1) and the second (PC2) principal components accounted together for 86.04%, 83.81% and 84.73% of the data variance, respectively for phylum, class, and order taxonomic levels.

### Microbial Community Richness and Diversity

The rarefaction curve plots, built based on the number of observed microbial groups vs. the number of sequences per sample, for both the four branches and the 11 individual samples, are reported in Online Resource 2 (a and b, respectively). Most curves tended to approach the saturation plateau, reinforcing the sufficiency of sequencing analysis, adequately representative of the investigated communities.

Alpha diversity estimation, using several metrics, is reported in Table 2. The total OTUs generated for each sample ranged from a maximum of 2127 to a minimum of 1323, whereas the average value of Good’s Coverage was 99.78%, indicating that the analysis well covers the microbial diversity in vermiculation samples. Chao1 richness estimator resulted between 1444.8 and 2313.3. Shannon and Simpson diversity indices presented similar estimates among the samples (around 7 and close to 1, respectively), except for P1, which presented the lowest values (5.78 and 0.87, respectively).

**Table 2:** Community richness and diversity estimated for each sample, using several alpha diversity metrics (Good’s Coverage, Chao1, Shannon, Simpson)

Sample	Operational taxonomic units	Good’s Coverage (%)	Chao1	Shannon	Simpson
A1	1431	99.88	1553.3	7.128	0.968
A2	1597	99.78	1780.1	7.265	0.978
A3	1712	99.81	1877.9	7.409	0.971
F1	2127	99.72	2313.3	7.739	0.977
F2	1963	99.60	2310.9	7.669	0.978
F3	1891	99.82	2009.6	7.228	0.952
F4	1730	99.73	1988.5	7.055	0.963
P1	1728	99.82	1909.3	5.784	0.874
T1	1929	99.80	2101.7	7.567	0.979
T2	1521	99.73	1705.6	7.149	0.969
T3	1323	99.88	1444.8	6.920	0.973

### Relationships Between Microbial Community and Geochemical Characteristics

Pearson’s correlation coefficients between microbial phyla and geochemical and mineralogical characteristics [17] of each vermiculation are shown in Table 3a. *Deferribacteres*, *Latescibacteria*, and *Nitrospirae* displayed positive correlations (0.74 < *r* < 0.77; *p* < 0.01), with organic C, P, and Mo, respectively. Unclassified *Archaea*, *Armatimonadetes*, and *Ignavibacteriae* were negatively correlated (− 0.61 < *r* < − 0.66; *p* < 0.05) with S. *Chloroflexi* were positively related with Ca, Mg, Sr, Ti, V, and Zn (0.60 < *r* < 0.65; *p* < 0.05), but negatively with C (*r* = − 0.66; *p* < 0.05). *Spirochaetes* were negatively correlated with Ca, Fe, Mg, Ti, Li, V, Cr, Zn, Cu, and quartz (with *r* values ranging from − 0.61 to − 0.67, and *p* < 0.05), and positively correlated with C and calcite (with correlation coefficients equal to 0.66 and 0.61, respectively and *p* values < 0.05). Furthermore, *N* showed a positive correlation with *Deferribacteres* phylum (*r* = 0.70; *p* < 0.05) and a negative relationship with *Elusimicrobia* phylum (*r* = − 0.67; *p* < 0.05), whereas *Verrucomicrobia* revealed a negative correlation with Co, K, Mn, and N (− 0.63 < *r* < − 0.65; *p* < 0.05). Among the *Archaea* phyla, *Woesearchaeota* was positively correlated with organic C, showing a correlation coefficient of 0.64 (*p* value < 0.05).

**Table 3:**
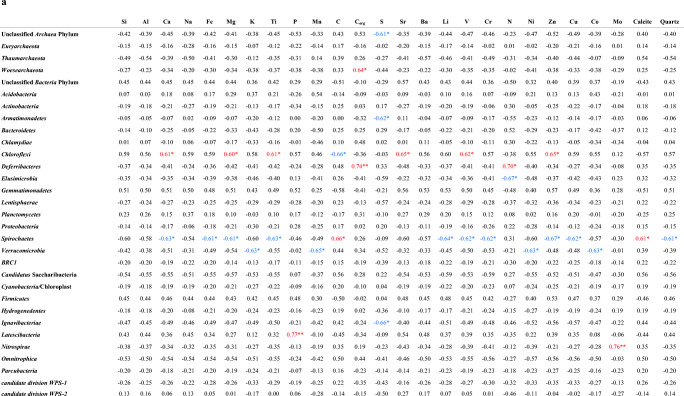

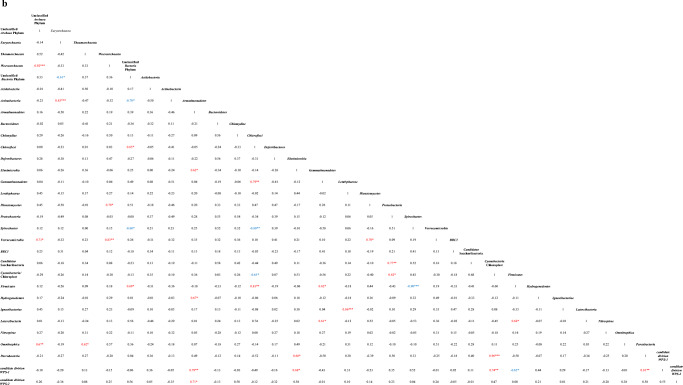
Pearson correlation matrices between microbial phyla and geochemical characteristics of the studied vermiculations (a) and among their biological properties (b). Significant (***p < 0.001; **p < 0.01; *p < 0.05) correlation coefficients are also highlighted (positive in red, negative in blue)

The correlation analysis results between microbial groups identified in the 11 studied vermiculations are reported in Table 3b. Positive correlations (*p* < 0.001) among several groups were observed: unclassified *Archaea* phylum with *Woesearchaeota* (*r* = 0.92), *Euryarchaeota* with *Actinobacteria* (*r* = 0.85), *Lentisphaerae* with *Ignavibacteriae* (*r* = 0.86), and *Cyanobacteria/*Chloroplast with *Parcubacteria* (*r* = 0.96). *Spirochaetes* is the only one displayed highly negative correlation with *Firmicutes* (*r* = − 0.90; *p* < 0.001). Moreover, *Chloroflexi* were positively correlated with *Gemmatimonadetes* (*r* = 0.79; *p* < 0.01), *Firmicutes* (*r* = 0.83; *p* < 0.01), and unclassified *Bacteria* phylum (*r* = 0.65; *p* < 0.05), but negatively with *Spirochaetes* (*r* = − 0.80; *p* < 0.01) and *Cyanobacteria/*Chloroplast (*r* = − 0.65; *p* < 0.05), whereas *Woesearchaeota* showed a positive correlation with *Verrucomicrobia* (*r* = 0.83; *p* < 0.01) and *Planctomycetes* (*r* = 0.70; *p* < 0.05). *Proteobacteria* displayed a positive correlation with *Candidatus* Saccharibacteria (*r* = 0.77; *p* < 0.01) and *Cyanobacteria/*Chloroplast (*r* = 0.62; *p* < 0.05); *candidate division WPS-1* was positively related with *Armatimonadetes* (*r* = 0.79; *p* < 0.01), *Cyanobacteria/*Chloroplast (*r* = 0.74; *p* < 0.01), *Parcubacteria* (*r* = 0.81; *p* < 0.01), and *Elusimicrobia* (*r* = 0.68; *p* < 0.05), but negatively correlated with *Firmicutes* (*r* = − 0.62; *p* < 0.05). Unclassified *Bacteria* phylum showed a negative correlation with *Actinobacteria*, *Spirochaetes*, and *Euryarchaeota* (− 0.60 < *r* < − 0.70; *p* < 0.05), but it was positively correlated with *Firmicutes* (*r* = 0.69; *p* < 0.05). *Latescibacteria* showed a positive correlation with *Gemmatimonadetes* (*r* = 0.61; *p* < 0.05) and *Firmicutes* (*r* = 0.68; *p* < 0.05), whereas *Armatimonadetes* with *Elusimicrobia*, *Hydrogenedentes* and *candidate division WPS-2*, with correlation coefficients ranging from 0.62 to 0.71 and *p* value < 0.05. *Omnitrophica* were positively correlated (*p* < 0.05) with unclassified *Archaea* phylum (*r* = 0.67) and *Thaumarchaeota* (*r* = 0.62). Finally, *Verrucomicrobia* highlighted a positive correlation with unclassified *Archaea* phylum (*r* = 0.73; *p* < 0.05), *Gemmatimonadetes* with *Firmicutes* (*r* = 0.62; *p* < 0.05), and *Elusimicrobia* with *Parcubacteria* (*r* = 0.68; *p* < 0.05).

**Fig. 3:**
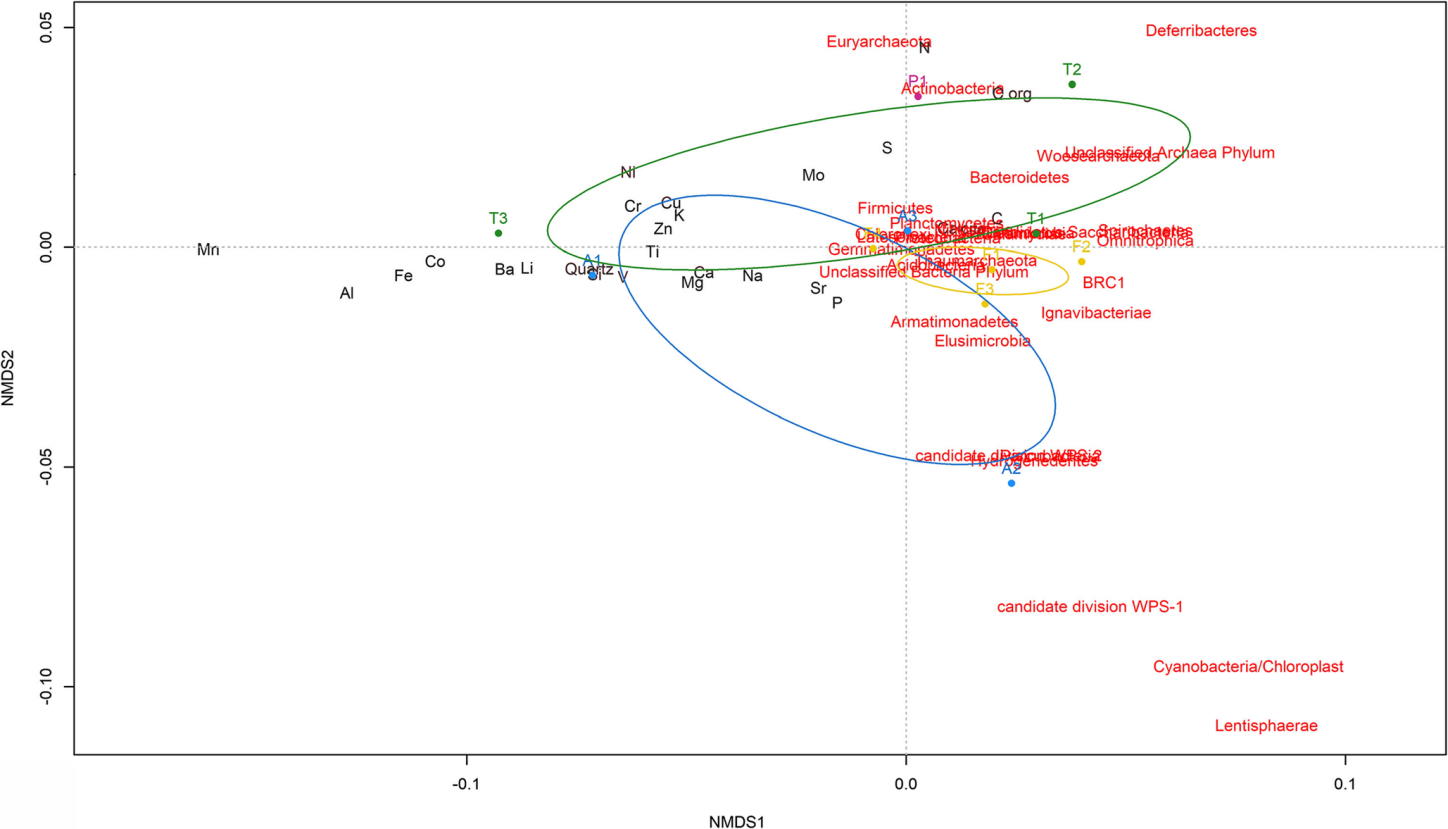
NMDS analysis, with confidence ellipses (*α* = 0.05) for the four branches [Active (A, blue), Fossil (F, yellow), Paradise (P, violet), and Tourist (T, green)], based on the total microbial community (red labels) and the geochemical characteristics (black labels) of the same vermiculations, as reported in Addesso et al. [17]

The NMDS biplot (Fig. 3), based on the microbiological and geochemical-mineralogical [17] characteristics of the analyzed vermicular deposits, showed a clear separation of the confidence ellipses grouping the Tourist and Fossil branches. The vermicular deposits from the active trail revealed intermediate characteristics, as highlighted by the partial overlapping of its confidence ellipse with the other two. Between the two most abundant minerals (calcite and quartz), calcite characterized the vermiculations from the four trails, whereas quartz mainly those of the Tourist and Active trails. Among the 24 elements (total Al, Ba, C, Ca, Co, Cr, Cu, Fe, K, Li, Mg, Mn, Mo, N, Na, Ni, P, S, Si, Sr, Ti, V, and Zn and organic C) analyzed, N, S, and organic C, mostly abundant in the vermiculations from the lightened trails (Paradise and Tourist), together with C, and to a lesser extent Mo, P, and Sr, showed a strong relationship with bacterial communities.

**Fig. 4:**
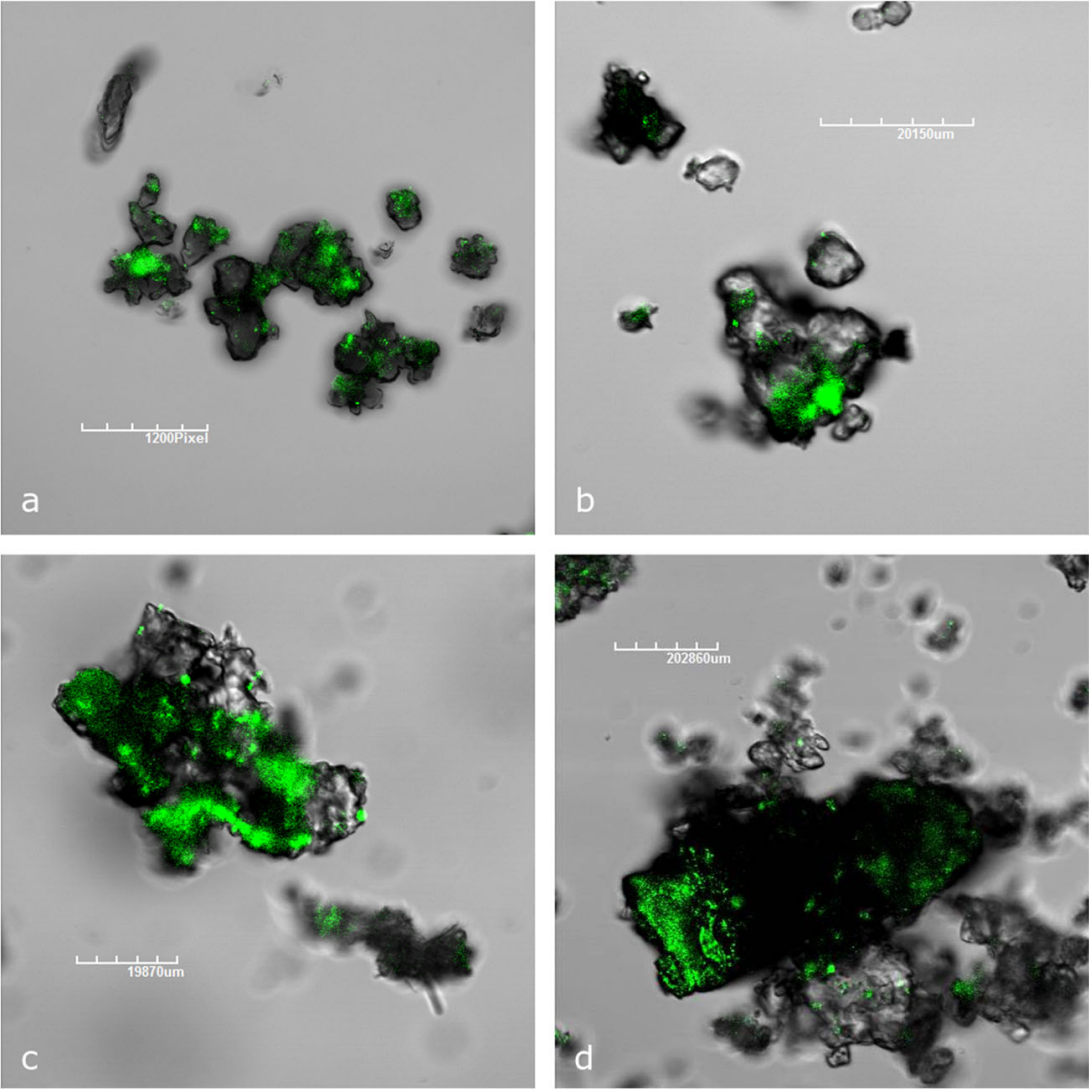
CLSM images of A4 (**a**, **b**), F1 (**c**), and T1 (**d**) vermiculation samples, showing the presence of microbial clusters (green-colored zones) dyed with SYBR Green staining

Confocal microscopic observations performed on samples A4 (Fig. 4a, b), F1 (Fig. 4c), and T1 (Fig. 4d) provided interesting information about the distribution and density of microbial communities (green-colored zones) on the mineral surface. As revealed by FESEM images (Online Resource 3), microbial structures were found mainly associated with clay minerals.

## Discussion

Although vermiculations represent a perfect substratum suitable for microbes, probably participating also to their formation as mediators of geochemical processes [10, 20], very little is known about the microbiota of such enigmatic deposits. In this context, our study provides, for the first time, an overview on the microbial life associated with vermiculations from non-sulfidic karst systems.

The NGS approach revealed a biodiversity comparable to those observed in several matrices from different caves [10, 19, 20, 33–35]. *Proteobacteria* (41.3–54.8%), represented (in decreasing order) by *Gamma-*, *Beta-*, *Alpha-*, and *Deltaproteobacteria* classes, was the dominant phylum, likely in relation to the wide ranges in metabolism and phenotype, offering the capability to degrade a broad spectrum of organic substrates and to adapt to and thrive in the hostile cave environment [2]. The presence of *Proteobacteria* is often associated with Fe-Mn deposits [36, 37], both chemical elements were observed in vermiculations from Pertosa-Auletta Cave [17], and mainly related to the geochemical characteristics of the substratum, as highlighted by the NMDS. The carotenoid-producing gammaproteobacterial *Xanthomonadales* order was also detected, typical of yellow-colored colonies found in caves [38, 39]. Among *Alphaproteobacteria*, the *Rhizobiales* order, represented by members able to fix nitrogen and to oxidize iron and manganese, and the *Rhodospirillales* order, equally participating to the nitrogen cycle, were observed. They are typical surficial microorganisms [2], but, as suggested by Lavoie et al. [40], their presence in caves can be related to the migration of microorganisms from above lying soils, and once in the cave they start an adaptation process to the new surrounding environmental conditions. Similar to vermiculations from the Pertosa-Auletta Cave, those in the sulfuric acid Fetida Cave (Apulia, Italy) showed a great abundance of *Proteobacteria* (44-46%), but with copious microbial communities belonging to *Deltaproteobacteria* (25%) and *Epsilonproteobacteria* (16%), respectively, dominated by *Desulfobacterales* and *Campylobacterales*, involved in the sulfur cycle [19, 20].

*Acidobacteria* represented the second most abundant phylum, whose genetic and metabolic diversity is comparable to the highly diverse *Proteobacteria* [41–43]. *Acidobacteria* often occur together with chemolithoautotrophic *Gammaproteobacteria*, suggesting a mutualistic association between them: *Acidobacteria* gain energy oxidizing the reduced organic compounds (chemoorganotrophy) obtained from *Proteobacteria* autotrophic metabolism, an ecological advantage in cave oligotrophic environments [44]. Only in the green P1 vermiculation, in the lightened Paradise branch, the most represented phylum after *Proteobacteria* was *Actinobacteria* (33.8%), with *Actinomycetales* order, clearly different from the other vermiculations (1.9–10.3%), confirmed also by PCAs. The abundance of *Actinobacteria* in this vermiculation is justified by their association with *Cyanobacteria*, a well-known relationship in lightened subterranean environments [45].

Commonly found in soil systems, *Actinobacteria* may have an important ecological role in biogeochemical cycles of cave ecosystems, mediating mineralization processes [34] and producing bioactive compounds, such as antimicrobials that allow the biotic control on other populations [46]. Cuezva et al. [7] demonstrated they are able to capture CO_2_ from the atmosphere and precipitate CaCO_3_ polymorphs, as shown in FESEM images of the same samples reported in Addesso et al. [17]. In particular, *Actinomycetales* are able to degrade recalcitrant organic compounds [47]. The relative humidity and availability of endo- and exogenous organic matter in the Paradise branch can explain their colonization success. In fact, here, the moisture reaches approximately 100%, due to the presence of an underground river nearby, promoting the proliferation of *Actinomycetes* [48]. Moreover, the Paradise trail is lit and frequented by tourists who, together with photoautotrophic communities growing close to artificial light systems, bring an important input in terms of organic compounds, facilitating heterotrophic populations, including *Actinomycetales* [49]*.*

The aerobic chemolithoautotrophic nitrite-oxidizing *Nitrospirae* and the anaerobic ammonium-oxidizing *Planctomycetes*, together with *Firmicutes*, able to reduce/oxidize sulfur, as well as chemo- or phototrophic *Chloroflexi*, were also found elsewhere in small amounts. Moreover, numerous less-represented taxonomic groups (with relative abundance < 1%) were observed in the 11 vermiculations investigated and their ecological role in this kind of ecosystem is still debated [2]. Among them, *Archaea* were also present, with the *Thaumarchaeota*, *Euryarchaeota*, and *Woesearchaeota* phyla, despite the archaeal DGGE profile highlighted a major number of bands in terms of core species richness. The same were found in considerable amount (< 4.3%) in Fetida Cave [20], where the relative abundances change (*Proteobacteria* > *Planctomycetes* > *Acidobacteria* > *Chloroflexi* > *Bacteroidetes* > *Actinobacteria* > *Nitrospirae*), likely due to the more extreme acidophilic environment, promoting the development of some bacterial groups rather than others. Despite the scarcity of knowledge about the archaeal group in cave ecosystems, it is well known that they give a relevant contribution to the global carbon nitrogen and sulfur cycles [22, 50, 51]. This may explain both the strong association between *Euryarchaeota* and N, and the relation of unclassified *Archaea* phylum groups, *Thaumarchaeota*, and *Woesearchaeota* with C and organic C highlighted by NMDS.

The Simpson index displays values close to 1 for all the samples, considering the dominant groups in the community and excluding the rare ones, indicating a low biodiversity and a high dominance. From the NGS results, it emerges that the dominant groups are unclassified already at the phylum level and this increases with the taxonomic level specificity. Values close to 7 were, instead, obtained for Shannon index, sensible also to the rare species, abundantly present in all the samples and certainly important from an ecological point of view.

Overall, geochemical and microbiological characteristics of the studied vermiculations differed among branches of the Pertosa-Auletta Cave, with the greatest differences observed between those from tourist and unvisited branches. Anyway, macroelements (C, N, S, and P), as well as the organic matter, were mostly abundant in the vermiculations from the Paradise and Tourist branches, highlighting the presence of more abundant biomass in lightened trails, where the photoautotrophs proliferate. In these samples, also Mo and Sr were more abundant, indicating that a specialized microbial community could have resulted from some microbial lineages able to oxidize minerals containing such elements [52]. However, F4 sample showed a high abundance of *Nitrospirae* phylum compared to the other vermicular deposits that displayed also a high correlation with molybdenum, probably due to its content in the membrane-associated enzyme of the nitrite-oxidizing system [53]. Furthermore, the higher content of organic C in vermiculations from Fossil and Tourist trails [17] may explain the major abundance of *Nitrospirae* in such locations, where the availability of ammonia by ammonificators can increase the presence of nitrites, in turn usable by nitrite-oxidizing *Nitrospirae* group bacteria [33]. From Pearson correlation analysis, several associations emerge between biological and geochemical properties, as well as among the taxonomic groups, especially the rarest, but they are not at all easy to explain, due to the lack of information about their biogeochemical role in the cave ecosystem [33].

Confocal microscopy images showed a localization of DNA only in specific sites, recognizable in the green zones. This was confirmed also by FESEM images, as reported in Addesso et al. [17], showing the clayey deposits always associated with biogenic filamentous material, not ruling out the possibility that the microbes can interact or influence their behavior and evolution in the environment [54, 55].

The findings of the present study support the theory formulated by Jones et al. [10], suggesting that microorganisms play an active role in vermiculation genesis, producing organic matter and secondary minerals, enriching the calcite matrix, trapping and binding sediment particles and dissolving, through etching or pitting, the rock. This may happen in different environments, from sulfuric acid to normal karst caves. However, beyond the biological evidences, the possibility of coexistence of several processes remains. For example, decalcification of rock walls, due to the dissolution processes caused by the acidity of seeping or condensation waters, can contribute to create the primordial calcite matrix [56–59]; thereafter, neutralization of electrical charges in the small particles, associated to wet-dry phenomena, can determine the different morphologies [16, 60]. Nevertheless, further studies are required to clarify to what extent some processes prevail over others, determining the variety of vermiculations described.

The present study, describing the microbiota present in the vermicular deposits of the Pertosa-Auletta Cave and its relationships with geochemistry of vermiculations, fills the gap characterizing these topics in karst caves. The analyses carried out indicate a certain diversity of biological communities living in vermicular deposits, with a considerable percentage of unclassified lineages, already at the phylum level, demonstrating once more that the underground ecosystem hosts still a high number of unknown taxa. *Proteobacteria* and *Acidobacteria* were the predominant phyla, as generally observed in such environments, whereas *Actinobacteria* showed an increased growth due to the high humidity conditions and the input of organic matter from the considerable presence of tourists in the show cave. The involvement of such communities in the biogeochemical cycles is indisputable and the highlighted biological evidences confirm a tight interaction between biotic and abiotic factors in the formation of vermiculations. The obtained findings represent a crucial step for the protection and conservation of such unique ecological niches, making still more intriguing the knowledge and comprehension pathway of vermiculations.

## Electronic supplementary material

ESM 1 (PDF 400 kb)

ESM 2 (PDF 402 kb)

ESM 3 (PDF 685 kb)

ESM 4 (PDF 72 kb)
