# Supplementary material for: Microbial Community Characterizing Vermiculations from Karst Caves and Its Role in Their Formation
Source: Microb Ecol. 2020 Nov 6;81(4):884–96. doi: 10.1007/s00248-020-01623-5 (PMC8062384; doi:10.1007/s00248-020-01623-5)

SUPPLEMENTARY MATERIAL

Microbial community characterizing vermiculations from karst caves and its role in their formation

Rosangela Addesso [1], Jose L. Gonzalez-Pimentel [2], Ilenia M. D'Angeli [3], Jo De Waele [3], Cesareo Saiz-Jimenez [4], Valme Jurado [4], Ana Z. Miller [2], Beatriz Cubero [4], Giovanni Vigliotta [1], Daniela Baldantoni [1]

[1] Department of Chemistry and Biology "Adolfo Zambelli", University of Salerno, Via Giovanni Paolo II, 132, 84084 Fisciano (SA), Italy; [2] HERCULES Laboratory, University of Évora, Largo Marques de Marialva 8, 7000-809 Évora, Portugal; [3] Department of Biological, Geological and Environmental Sciences, University of Bologna, Via Zamboni, 67, 40126 Bologna, Italy; [4] Instituto de Recursos Naturales y Agrobiología de Sevilla, IRNAS-CSIC, Av. Reina Mercedes, 10, 41012, Sevilla, Spain

* Corresponding author: Rosangela Addesso; email: raddesso@unisa.it

Online Resource 1 Archaeal (A) and bacterial (B) 16S rRNA gene-DGGE profiles of sampled vermiculations from the Active (blue), Fossil (yellow), Paradise (violet) and Tourist (green) branches.

a

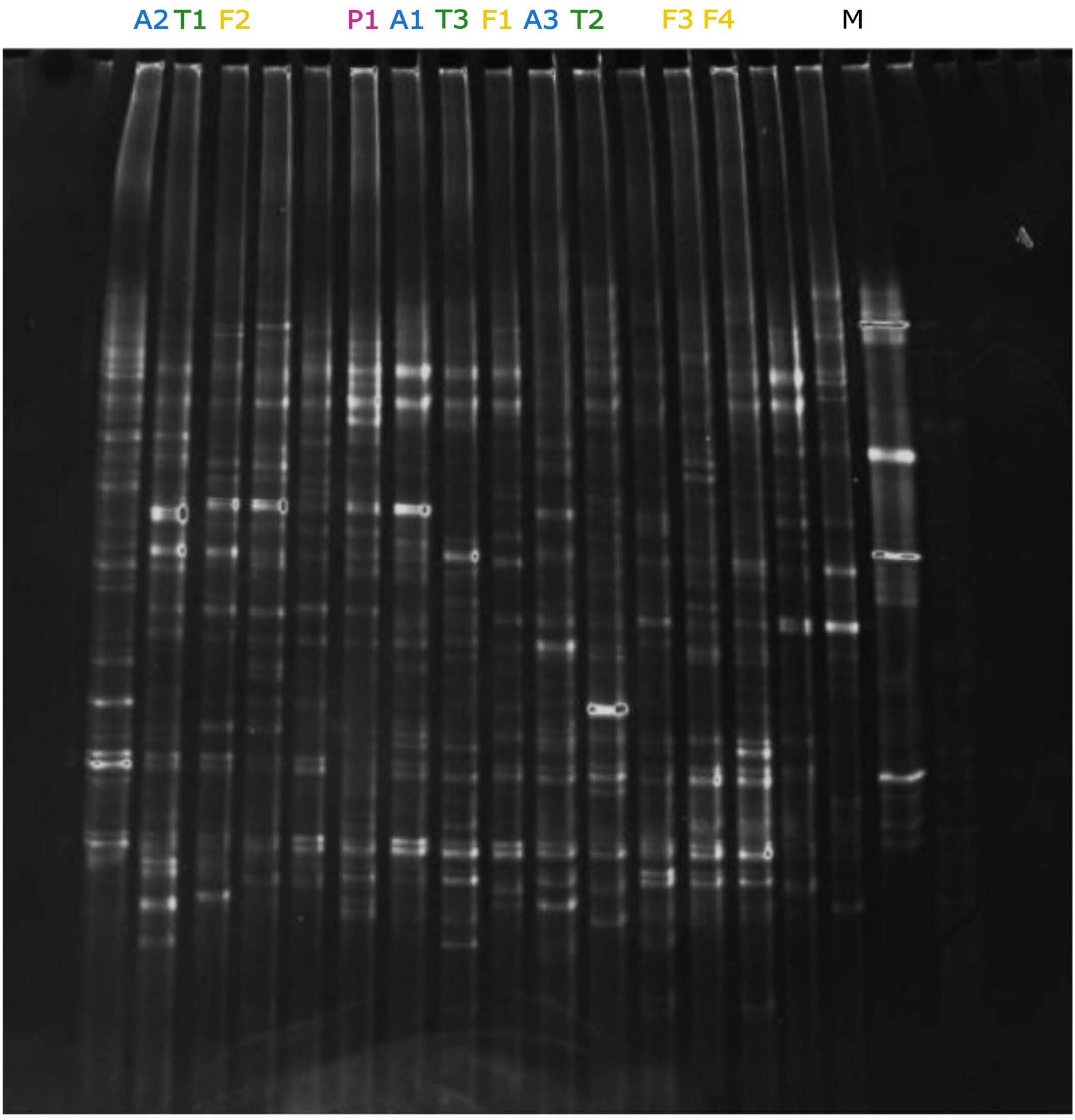

b

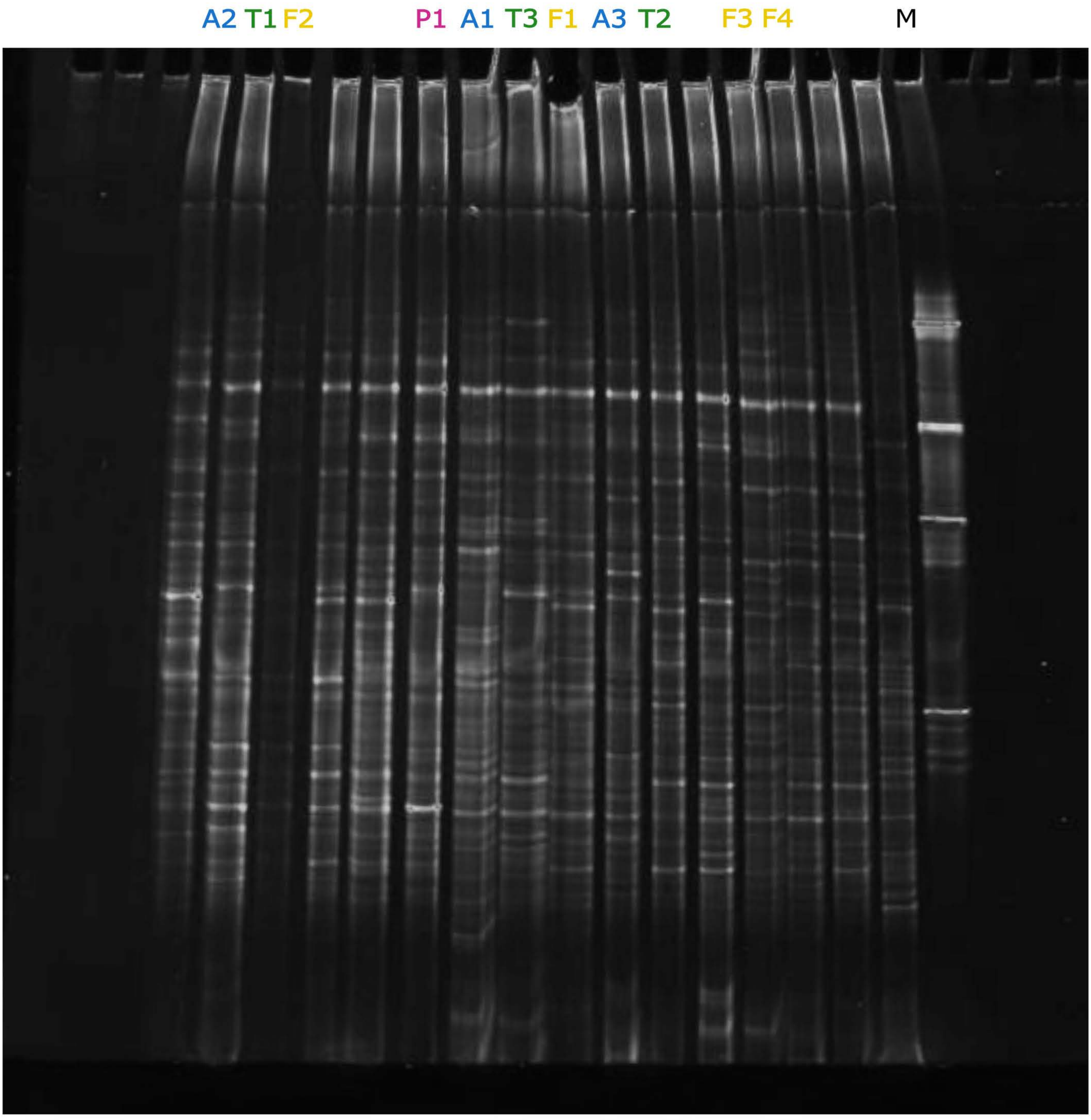

Supplement: Supplementary file 1 — (PDF 400 kb) [file 248_2020_1623_MOESM1_ESM.pdf]
