# Supplementary material for: Microbial Community Characterizing Vermiculations from Karst Caves and Its Role in Their Formation
Source: Microb Ecol. 2020 Nov 6;81(4):884–96. doi: 10.1007/s00248-020-01623-5 (PMC8062384; doi:10.1007/s00248-020-01623-5)
Supplement: Supplementary file 4 — (PDF 72 kb) [file 248_2020_1623_MOESM4_ESM.pdf]

SUPPLEMENTARY MATERIAL

Microbial community characterizing vermiculations from karst caves and its role in their formation

Rosangela Addesso [1], Jose L. Gonzalez-Pimentel [2], Ilenia M. D'Angeli [3], Jo De Waele [3], Cesareo Saiz-Jimenez [4], Valme Jurado [4], Ana Z. Miller [2], Beatriz Cubero [4], Giovanni Vigliotta [1], Daniela Baldantoni [1]

[1] Department of Chemistry and Biology "Adolfo Zambelli", University of Salerno, Via Giovanni Paolo II, 132, 84084 Fisciano (SA), Italy; [2] HERCULES Laboratory, University of Évora, Largo Marques de Marialva 8, 7000-809 Évora, Portugal; [3] Department of Biological, Geological and Environmental Sciences, University of Bologna, Via Zamboni, 67, 40126 Bologna, Italy; [4] Instituto de Recursos Naturales y Agrobiología de Sevilla, IRNAS-CSIC, Av. Reina Mercedes, 10, 41012, Sevilla, Spain

* Corresponding author: Rosangela Addesso; email: raddesso@unisa.it

Online Resource 4 PCR thermal programs with specific primers used for Prokarya and Eukarya.

<table>
<tr><th>Domain</th><th>Kingdom</th><th>Primer</th><th>Sequence</th><th colspan="2">Program</th></tr>
<tr><td rowspan="8">Prokarya</td><td rowspan="4">Bacteria</td><td rowspan="2">616F</td><td rowspan="2">5′-AGAGTTTGATYMTGGCTCAG-3′</td><td colspan="2">94 °C - 2 min</td></tr>
<tr><td rowspan="2">94 °C - 20 sec
55 °C - 20 sec
72 °C - 2 min</td><td rowspan="2">35 cycles</td></tr>
<tr><td rowspan="2">1510R</td><td rowspan="2">5′-GGCTACCTTGTTACGACTT-3′</td></tr>
<tr><td colspan="2">72 °C - 10 min</td></tr>
<tr><td rowspan="4">Archaea</td><td rowspan="2">109F</td><td rowspan="2">5′-ACKGCTCAGTAACACGT-3′</td><td colspan="2">95 °C - 5 min</td></tr>
<tr><td rowspan="2">95 °C – 1 min
55 °C – 1 min
72 °C - 3 min</td><td rowspan="2">25 cycles</td></tr>
<tr><td rowspan="2">915R</td><td rowspan="2">5′-GTGCTCCCCCGCCAATTCCT-3′</td></tr>
<tr><td colspan="2">72 °C - 10 min</td></tr>
<tr><td rowspan="8">Eukarya</td><td rowspan="4">Eukarya</td><td rowspan="2">EukA</td><td rowspan="2">5′-AACCTGGTTGATCCTGCCAGT-3´</td><td colspan="2">95 °C - 2 min</td></tr>
<tr><td rowspan="2">95 °C - 15 sec
50 °C - 15 sec
72 °C - 2 min</td><td rowspan="2">35 cycles</td></tr>
<tr><td rowspan="2">EukB</td><td rowspan="2">5′-TGATCCTTCTGCAGGTTCACCTAC-3´</td></tr>
<tr><td colspan="2">72 °C - 10 min</td></tr>
<tr><td rowspan="4">Fungi</td><td rowspan="2">ITS1</td><td rowspan="2">5´-TCCGTAGGTGAACCTGCGG-3´</td><td colspan="2">94 °C - 2 min</td></tr>
<tr><td rowspan="2">94 °C – 1 min
50 °C – 1 min
72 °C - 1 min</td><td rowspan="2">35 cycles</td></tr>
<tr><td rowspan="2">ITS4</td><td rowspan="2">5´-TCCTCCGCTTATTGATATGC-3´</td></tr>
<tr><td colspan="2">72 °C - 5 min</td></tr>
</table>
